# A Comparative Study between the Outcome of Primary Repair versus Loop Ileostomy in Ileal Perforation

**DOI:** 10.1155/2014/729018

**Published:** 2014-03-27

**Authors:** Sushil Mittal, Harnam Singh, Anand Munghate, Gurpreet Singh, Anjna Garg, Jyoti Sharma

**Affiliations:** ^1^Department of Surgery, Government Medical College, Patiala 147001, India; ^2^Department of Pathology, Pandit Bhagwat Dayal Sharma Post Graduate Institute of Medical Sciences, Rohtak 124001, India

## Abstract

*Introduction*. Ileal perforation peritonitis is a common surgical emergency in the Indian subcontinent and in tropical countries. It is reported to constitute the fifth common cause of abdominal emergencies due to high incidence of enteric fever and tuberculosis in these regions. *Methods*. Sixty proven cases of ileal perforation patients admitted to Surgical Emergency were taken up for emergency surgery. Randomisation was done by senior surgeons by picking up card from both the groups. The surgical management was done as primary repair (group A) and loop ileostomy (group B). *Results*. An increased rate of postoperative complications was seen in group A when compared with group B with 6 (20%) patients landed up in peritonitis secondary to leakage from primary repair requiring reoperation as compared to 2 (6.67%) in ileostomy closure. A ratio of 1 : 1.51 days was observed between hospital stay of group A to group B. *Conclusion*. In cases of ileal perforation temporary defunctioning loop ileostomy plays an important role. We recommend that defunctioning ileostomy should be preferred over other surgical options in cases of ileal perforations. It should be recommended that ileostomy in these cases is only temporary and the extra cost and cost of management are not more than the price of life.

## 1. Introduction

Gastrointestinal perforations have been surgical problem since the time immortal. Scientists have found evidence of gastrointestinal perforations in Egyptian mummies. Perforation is said to occur once a pathology which extends through the full thickness of the hollow viscus leading to peritoneal contamination with intraluminal contents. Perforation can occur anywhere in the gastrointestinal tract starting from oesophagus to the rectum [1].

Ileal perforation peritonitis is a common surgical emergency in the Indian subcontinent and in tropical countries. It is reported to constitute the fifth common cause of abdominal emergencies due to high incidence of enteric fever and tuberculosis in these regions. Despite the availability of modern diagnostic facilities and advances in treatment regimes, this disease has an abrupt onset and a rapid downhill course with a high mortality if not treated [2, 3].

Various causes of nontraumatic ileal perforation include bacterial infections (salmonella, yersinia, and tuberculosis), viral infections (cytomegalovirus, human immunodeficiency virus), fungal infection (histoplasma), parasitic infections (A. lumbricoides, E. vermicularis, and E. histolytica), and others (Wagener's granulomatous and drugs (Nonsteroidal anti-inflammatory drugs, e.g., aspirin, paracetamol, mefenamic acid, Ibuprofen, etc.)). In a significant number of cases the cause of perforation is not known and it is called nonspecific ileal perforation. The perforation causes gram-negative aerobic and anaerobic infection leading to peritonitis [4].

Various operative procedures were advocated by different authors, such as the following:

- simple primary repair of perforation [5];
- repair of perforation with ileotransverse colostomy [6];
- primary ileostomy [7, 8];
- single layer repair with an omental patch [9];
- resection and anastomosis [10].

Even with such a variety of procedures, ileal perforation still has a high rate of morbidity and mortality. The aim of the present study is to evaluate the outcome of primary repair versus loop ileostomy in cases of ileal perforation by comparing them in terms of postoperative morbidity, mortality and cost-effectiveness, and complications and to find out the ideal procedure. The study will help to establish the criteria for instituting the management modality according to presentation and severity of the disease and the outcome of these procedures. Effective management of the disease will help in decreasing morbidity and mortality associated with the disease.

## 2. Material and Method

This comparative study was conducted in the Department of General Surgery, Government Medical College and Rajindra Hospital Patiala. Sixty patients admitted to Surgical Emergency with acute abdomen were selected for the study. There were not any preoperative selection criteria; the cases which were proven to be cases of perforation peritonitis on the basis of investigations and clinical examination were taken for study and considered for comparative study if laparotomy diagnosed to be case of ileal perforation. These patients were taken up for emergency surgery after resuscitation, and an informed consent was taken. The antibiotics were given in both groups after admission to hospital and before surgery with 3rd generation cephalosporin (cefotaxime, ceftazidime, ceftriaxone, etc.) and metronidazole. These patients were divided into two groups group A and group B. Randomisation was done by senior surgeons by picking up card from both the groups. The surgical management was done as primary repair (group A) and loop ileostomy (group B); comparative study was done between both procedures. All operations were done by group of three experienced surgeons and they all performed the same technique. All the procedures were carried with hand sewn method. In group A primary closure was done in two layers, the inner layer closed with 3-0 poly glycolic acid (vicryl) and outer layer closed with silk 3-0. In group B loop ileostomy was done. Postoperative complications in each group like wound infection, wound dehiscence, intra abdominal abscess, stricture of anastomosis site, faecal fistula, peritonitis, septicemia, ileostomy related complications, paralytic ileus, intestinal obstruction and death and so forth are evaluated.

## 3. Results

**Figure 1:**
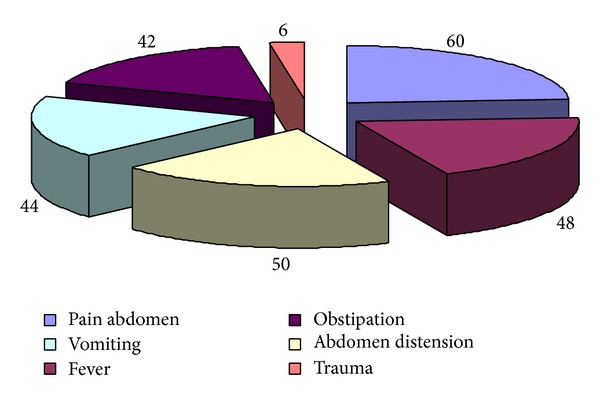
Clinical presentation in study group.

**Figure 2:**
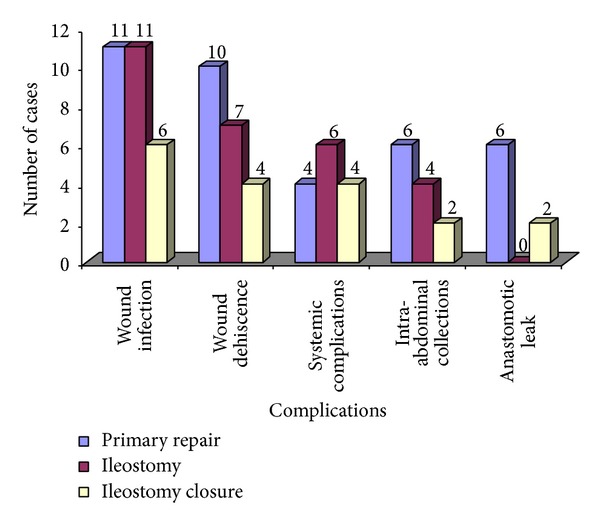
Complications in primary repair, ileostomy, and ileostomy closure.

During the 12-month period of study, 60 patients with ileal perforation were studied. Ileal perforations were most commonly observed in third and fourth decade of life with males more commonly affected (Male : Female: 6.5 : 1) (Table 1). Pain abdomen was the most common clinical presentation (100%) followed by fever, abdominal distension, vomiting, and obstipation (Figure 1). Time since perforation was within 12 hour in 3 cases, between 12 and 24 hour in 23 cases, between 24 and 48 hour in 18 cases, 48 and 72 hour in 7 cases, between 72 and 96 hour in 8 cases, and between 96 and 120 hour in 2 cases. Most of the patients (83.33%) presented within 72 hours of perforation and most cases were operated within 12 hours of presentation after adequate resuscitation. The average duration of fever was 7.2 days whereas in patients with typhoid perforation, the average duration of fever was 10.41 days ranging from 1 day to 25 days. Fever preceded the abdominal symptoms in these patients. In all the cases in study group, the etiology of perforation was typhoid, circular perforation at antimesenteric border (36.67%), nonspecific (35%), tuberculosis, elliptical perforation at antimesenteric border (18.33%), and trauma (10%). There was not any case having malignant cause of ileal perforation. Complications: wound infection was the commonest complication (36.67%). It was present in about 11 (36.67%) cases each in patients having undergone primary repair of perforation and patients having undergone ileostomy. Ileostomy related complications occurred in 19 patients (63.33%). Peristomal skin excoriation was the most common ileostomy related complication in 10 patients (33.33%) followed by weight loss in 4 (13.33%), retraction in 4 (13.33%), fluid and electrolyte imbalance in 3 (10%), and prolapse in 1 (3.33%). Ileostomy closure related complications occurred in 7 patients (11.67%), wound infection in 6 (20%), anastomotic leak in 2 (6.67%), intra-abdominal collections in 2 (6.67%), wound dehiscence in 4 (13.33%), and reoperations in 2 (6.67%) (Figure 2). The complications between the two groups were statistically significant with *P* value 0.026, with chi square test *χ* ^2^ value 9.24 with degree of freedom Df 3. The average duration of hospital stay in patients having undergone primary closure was 14.3 days compared to 21.53 days in patients with ileostomy, which included ileostomy closure. The average duration of ileostomy before closure was 208.1 days (about 3.6 months). Only two patients having DM and outcome of these patients were good and remaining 58 patients were having no any comorbidities. In all the cases the biopsy was sent and histopathological examination was done and found to be typhoid enteritis 12 (20%), tubercular 6 (10%), and nonspecific inflammation in the remaining 42 (70%) cases.

## 4. Discussion

Ileal perforation peritonitis is a common surgical emergency in the Indian subcontinent and in tropical countries. It is reported to constitute the fifth common cause of abdominal emergencies due to high incidence of enteric fever and tuberculosis in these regions. Despite the availability of modern diagnostic facilities and advances in treatment regimes, this disease has an abrupt onset and a rapid downhill course with a high mortality if not treated [2, 3].

Onset of symptoms and time of presentation in hospital are important prognostic factors. An early presentation holds a good prognosis even with primary repair of perforation. Unfortunately, in developing countries like India, the presentation to hospital is usually late with fully blown peritonitis; some cases may present with septicemia and multiorgan [11]. Various operative procedures were advocated by different authors, such as simple primary repair of perforation [5], repair of perforation with ileotransverse colostomy [6], primary ileostomy [7, 8], single layer repair with an omental patch [9], and resection and anastomosis [10]. In our study we compare the outcome of primary closure versus loop ileostomy in ileal perforation in terms of complications and know ideal procedure between these two groups.

Small bowel perforations most commonly affect the young in the prime of their life. In the present study male preponderance was found with male to female ratio of 6.5 : 1 which is the higher side of the ratio 3 : 1 reported by Wani et al. [12], 4 : 1 reported by Adesunkanmi et al. [13] and Talwar et al. [14], 6.4 : 1 reported by Beniwal et al. [15], and 6.5 : 1 reported by Prasad et al. [6]. The mean age was 36.46 years with range of 15–70. The mean age was higher in our study as children below 12 years of age were excluded. Majority of patients were in the age group 21–40 years (53.33%). The peak incidence for age was in the fourth decade followed by third decade [12–15].

The study gives insight into contemporary causes of nontraumatic perforation of the small intestine in this part of the world on the basis of Widal reaction, operative findings, and histopathological examination. Typhoid remains the major identifiable cause of small bowel perforation (36.67%), the second being tubercular perforation (18.33%). In a large proportion of cases (35%), the underlying cause was not identified and histopathological analysis revealed nonspecific inflammation. Traumatic cause of ileal perforation was found to be in 10% of cases. The causes for nontraumatic ileal perforation were enteric fever (62%), nonspecific inflammation (26%), obstruction (6%), tuberculosis (4%), and radiation enteritis (1%) as reported by Wani et al. [12]. Nadkarni et al. found 56.6% nonspecific causes, followed by typhoid perforation (25%) and tubercular perforation (9.3%) [1].

The morbidity was higher in patients who underwent ileostomy as compared to patients who underwent primary repair in our study. There was no mortality in our study compared to 28% in other studies. However mortality was unrelated to type of operation performed. Wound infection was the most common postoperative complication, about 36.67% each in group I and group II, followed by wound dehiscence, intra-abdominal collections, systemic complication, and anastomotic leak. Eight out of 30 cases of ileal perforation proceed with primary repair having gross fecal contamination, out of eight; two cases had complication like anastomotic leak and subsequently reoperation was done; in one case ileostomy was done and in another case primary repair was done. The complications between the two groups were statistically significant with *P* value 0.026, with chi square test *χ* ^2^ value 9.24, and with degree of freedom Df 3, which is in accordance with previous studies (*P* value < 0.05) [12, 13].

The other complications in group II were related to ileostomy which hampered quality of life and significantly added to morbidity in these patients. Ileostomy related complications occurred in 18 patients (60%) and closure related complications occurred in 7 patients (23.33%). Ileostomy related complication rate in our study was higher in previous studies as reported by Bakx et al. [16]. Peristomal skin excoriation occurred in 33.33% of the patients and this was the most frequently recognized early complication [17]. It was followed by weight loss and retraction (13.33%), fluid and electrolyte imbalance (10%), and prolapsed (3.3%). The average duration of hospital stay of the patients in group I was 14.23 days and in group II 10.7 and 10.83 for ileostomy closure. The hospital stay of the patients was slightly longer in case of ileostomy (21.53 days) in comparison with primary repair (14.23 days).

This study also highlights the life-saving role of salvage loop ileostomy for postoperative intestinal leakage in cases of primary repair of perforation. The authors recommend that whenever intestinal leakage is suspected in the postoperative period, urgent exploratory laparotomy must be undertaken and the continuing peritoneal contamination should be controlled by exteriorizing the site of intestinal leak as loop ileostomy.

It is difficult to make a statement, whether ileostomy is better than primary repair of perforation because of small incidence of these complications and small size of our study and it need to be evaluated further with large number of patients; however for a single perforation, primary closure of the perforation was the procedure of choice where there is low volume of peritoneal contaminant.

## 5. Conclusion

Temporary defunctioning loop ileostomy in cases of ileal perforation plays an important role in reducing the incidence of complications like faecal fistula. This helps reduce mortality in patients undergoing surgery for ileal perforations. Ileostomy-specific complications, however, increase the postoperative stay of the patient. These complications can be reduced, if not outright eliminated, by proper fashioning of the stoma and provision of adequate nursing care of the stoma. We recommend that defunctioning loop ileostomy should be preferred over other surgical options in cases of ileal perforations in randomised study. It should be recommended that ileostomy in these cases is only temporary and the extra cost and cost of management are not more than the price of life.

## Figures and Tables

**Table 1:** Age distribution in both the groups.

Age group (in years)	Group A		Group B	
	Number of cases	% age	Number of cases	% age
10–20	3	10	4	13.33
21–30	7	23.34	8	26.66
31–40	10	33.33	7	23.34
41–50	4	13.33	7	23.34
51–60	4	13.33	4	13.33
61–70	2	6.67	0	0
Total	**30**	**100**	**30**	**100**
Range	15–70		16–60	
